# Developing theory and practice: Creation of a Community of Practice through Action Research produced excellence in stroke care

**DOI:** 10.3109/13561820.2010.483024

**Published:** 2010-08-27

**Authors:** Cherry Kilbride, Lin Perry, Mary Flatley, Emma Turner, Julienne Meyer

**Affiliations:** 1 Brunel University, Physiotherapy, Centre for Research in Rehabilitation, Uxbridge, UK; 2 University of Technology, Faculty of Nursing, Midwifery & Health, Sydney, Australia; 3 City University, School of Community & Health Sciences, London, UK; 4 Royal Free Hampstead NHS Trust, Neurosciences, London, UK

**Keywords:** Community of Practice, Action Research, stroke, Stroke Unit, evidence-based healthcare, evidence-based practice, inter professional team

## Abstract

Much emphasis is placed on expert knowledge like evidence-based stroke guidelines, with insufficient attention paid to processes required to translate this into delivery of everyday good care. This paper highlights the worth of creating a Community of Practice (CoP) as a means to achieve this. Drawing on findings from a study conducted in 20002002 of processes involved in establishing a nationally lauded high quality Stroke Unit, it demonstrates how successful development of a new service was linked to creation of a CoP. Recent literature suggests CoPs have a key in implementing evidence-based practice; this study supports this claim whilst revealing for the first time the practical knowledge and skills required to develop this style of working. Findings indicate that participatory and democratic characteristics of Action Research are congruent with the collaborative approach required for developing a CoP. The study is an exemplar of how practitioner researchers can capture learning from changing practice, thus contributing to evidence-based healthcare with theoretical and practical knowledge. Findings are relevant to those developing stroke services globally but also to those interested in evidence-based practice.

## INTRODUCTION

Stroke has the second highest death rate in the world (World Health Organization [WHO]) and is a major source of long term disability (American Heart Association, 2009), so it is essential that stroke patients receive the best possible care. Patients treated on a Stroke Unit (SU) where interventions are delivered by a specialist interprofessional team with expertise in stroke have better outcomes than those managed elsewhere (Intercollegiate Stroke Working Party [ISWP], 2008; Stroke Unit Trialists' Collaboration [SUTC], 2007). Comprehensive evidence-based stroke care is not yet global (WHO, 2009). Even the UK with universal coverage by a public health system, inequalities continue despite national stroke policies, evidence-based stroke guidelines and interprofessional audits to benchmark quality of stroke care (Department of Health [DH], 2007, 2008; Hoffman et al., 1998, 1999, 2002, 2004, 2006, 2008; ISWP, 2008; National Audit Office, 2010). Substantial gains have been made in recent years but much remains unknown about how good SU care is developed on the ground and why some SUs are more successful than others (DH, 2007; SUTC, 2007). Expert knowledge is only part of the story; if the challenge to provide best care for stroke is to be met, more attention must be paid to practical knowledge.

In countries where stroke services are well-developed, like the UK, care is commonly delivered within geographically discrete units, a set-up showing better outcomes than other models. Nonetheless essential elements remain poorly understood (Langhorne & Pollock, 2002).

**Figure 1:**
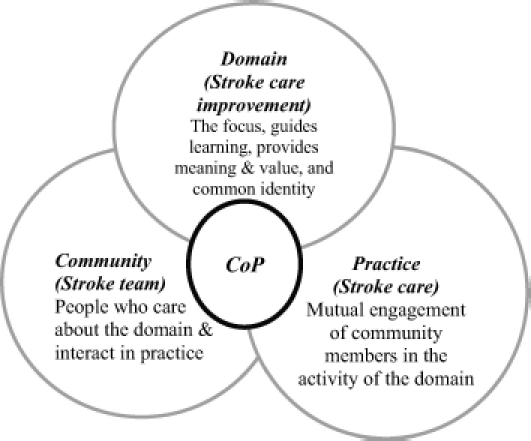
The three inter-related key elements of a Community of Practice.

Communities of Practice (CoPs) have been suggested as one mechanism to promote quality care. Essentially, a CoP comprises a group of people who work along collegial lines, share a concern or passion for something they do, and through regular interaction learn together how to do it better (Wenger et al., 2002). CoPs have three inter-related elements: domain, community and practice (Figure 1). They are rooted in a social theory of learning, hence embedding learning within relationships and social participation. Little is known about how CoPs are developed in practice, especially interprofessional CoPs (Dopson & Fitzgerald, 2006); this study explains how excellence in stroke care was achieved through creating one.

## METHODS

### Research setting

This study took place during 2000-2002 in a large UK NHS London teaching hospital with an established reputation for excellence. Nonetheless, care for stroke patients was uncoordinated and fragmented across 18 wards. The drive to improve stroke care came from a self-established group of nursing, therapy and medical staff, who were instrumental in service development action cycles congruent with the research design (Kilbride et al., 2005; see Table I and Results section for activities supporting SU development). At project commencement two wards were opened for the SU, with staff drafted to cover them. The project lead (CK) remained Head of Physiotherapy part-time; her “insider” role is discussed elsewhere (Kilbride et al., 2005). Supervisory support was provided by co-authors JM, LP and MF.

**Table I:** Operational infrastructure components implemented during Stroke Unit development process.

Joint intervention sessions	Senior management meetings
Goal planning	Board rounds
Case coordinator system	Ward rounds
Team meetings	Staff rotations
Structured assessments	Patient information group
SU joint progress meetings	Interprofessional education group
Development meetings	Timetables
Family meetings	Information whiteboards
Guidance on common stroke problems	Interprofessional documentation

### Aims and design

This case study (Kilbride et al., 2005; Kilbride, 2007) examined processes involved in developing a new SU and identified key factors influencing outcomes. Action Research was selected as the most appropriate methodology given the impetus of the study arose from clinicians and managers with dual objectives of effecting changes in clinical practice and developing knowledge of the processes. There were three study phases: exploration, innovation and evaluation (Table II). The postscript phase refers to the period following the end of formal study and withdrawal of CK, indicating unit performance after conclusion of the “project” phase of service development.

**Table II:** Summary of data collection in study phases.

*(1) Exploration phase data sets* Focus groups *N*=8 Pre-implementation NSSA 1998 Pre-implementation NSSA 1999 Reflective field notes recorded daily Meeting minutes	*(3) Evaluation phase data sets* Semi-structured interviews *N*=28 Reflective field notes recorded daily Post-implementation NSSA 2002 Meeting minutes
*(2) Innovation phase data sets* Reflective field notes recorded daily Meeting minutes	*(4) Postscript data sets* NSSA 2004 NSSA 2006 NSSA 2008

NSSA, National Sentinel Stroke Audit.

### Participants

Workforce movement was reflective of inner-city employment; during the study, excluding rotational doctors and students, 39 staff left and 40 joined the SU team. All staff involved with service delivery participated in development processes and were the focus of participant observation field notes. It was not feasible to engage all staff in all forms of data collection so data were gathered from a convenience sample of 40 staff across the range of those engaged with the project (Table III). Patient representation was obtained for the steering group.

**Table III:** Details of staff study participants.

Staff group	No.	Staff group	No.	Staff group	No
Nurses	22	Speech & language therapists	2	Dietitian	1
Physiotherapists	10	Pharmacist	2	Social worker	1
Occupational therapists	8	Discharge coordinator	2	Clinical psychologist	1
Doctors	5	Red Cross volunteer	2	Domestic staff	1
Therapy assistants	5	Ward clerk	1	Friends of the hospital	1
Healthcare assistants	4	Stroke coordinator	1	Catering manager	1
Trust managers	3	Volunteer service representative	1		

### Data collection

Data generating methods incorporated individual interviews, focus groups, and reflective field notes based on participant observations. Methods chosen to maximize the range of professional perspectives captured included policy documents, meeting minutes, individual viewpoints, joint constructions revealed by focus groups, and snapshots of practice from reflective field notes. Qualitative data were collected by a single researcher (CK). National Sentinel Stroke Audit (NSSA) data were used to track outcomes of change processes over time (1998-2008). These data are referred to here to demonstrate that development processes resulted in a high-performance SU. Table II lists data collected during each phase.

### Data analysis

Qualitative data were analysed thematically using a process of Immersion/Crystallization. In this systematic, iterative process the researcher read and re-read to immerse herself in the texts, created notes and coded text with intuitive interpretations. Each re-reading sought evidence for congruent and different perspectives (Borkan, 1999). Progress was regularly presented and discussed with co-authors who also examined selected transcripts. Textual interpretation was by consensus.

Quantitative data were extracted from six NSSAs, pre-and post-implementation (Hoffman et al., 1998, 1999, 2002, 2004, 2006, 2008), and analysed descriptively; these data have been reported elsewhere *(Health Service Journal* [HSJ], 2005; Kilbride, 2007).

### Ethical considerations

Ethical approval was obtained from the Local Research Ethics Committee, with on-going negotiation of participant involvement throughout.

## RESULTS

Process findings occurred alongside consistent improvement in outcomes as measured by successive NSSAs (Hoffman et al., 2002, 2004, 2006, 2008). These included organizational targets such as SU admission and waiting times, and process indicators like assessments and care planning. The stroke team received a national award in 2005 in recognition of moving the service from the bottom 5% of NSSA scores to become the top scoring service within four years (HSJ, 2005). Findings reported here reflect processes involved in this achievement.

### Key emergent process findings

**Figure 2:**
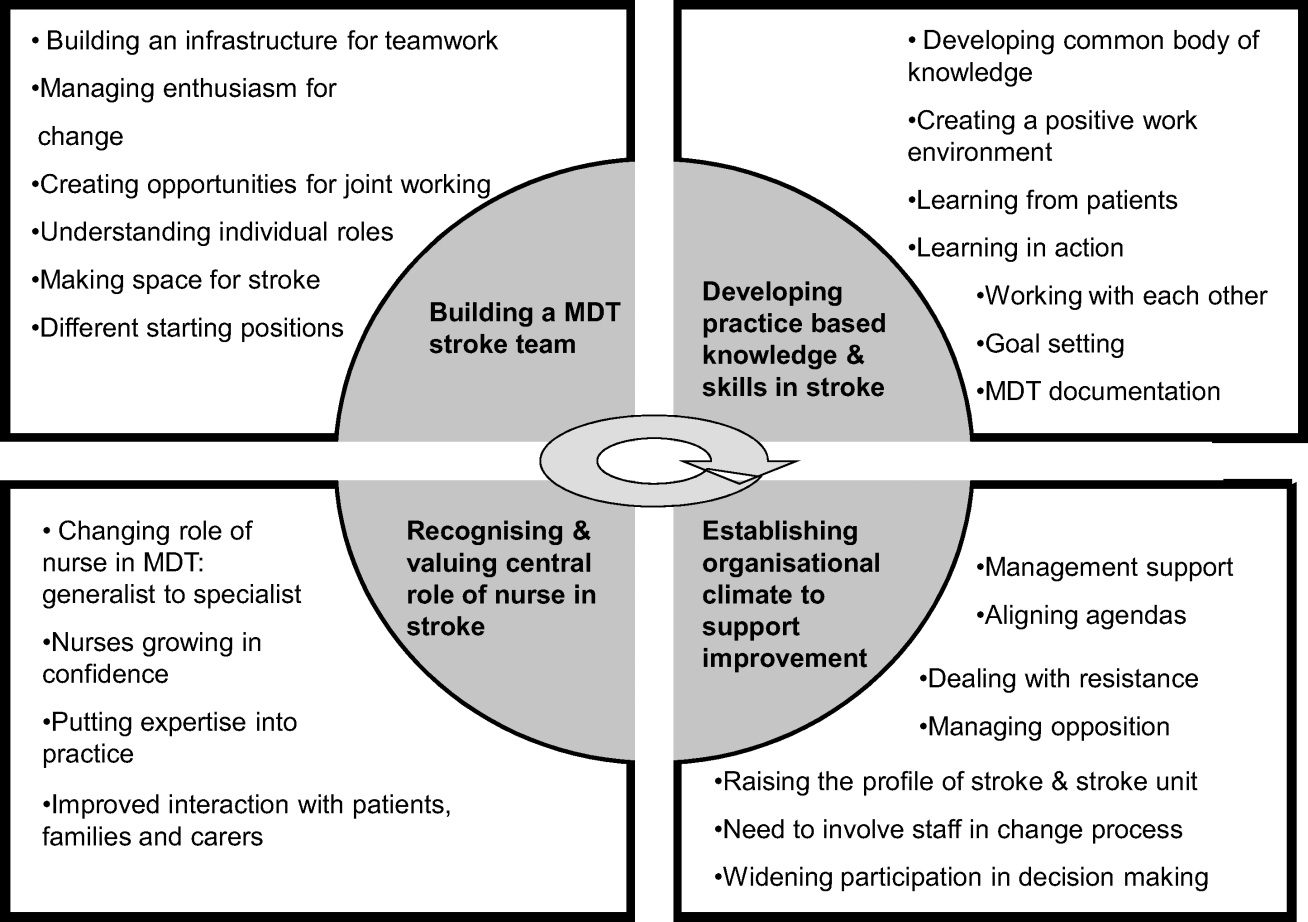
Summary of key emergent process findings.

Four key inter-related themes emerged from the data: the importance of (i) building an interprofessional stroke team; (ii) developing practice-based knowledge and skills in stroke care; (iii) valuing the central role of the nurse in stroke care, and (iv) creating an organizational climate for supporting improvement. Each impacted the others, and development occurred in a non-linear fashion. Themes and sub-themes are summarized in Figure 2 and detailed in Kilbride et al. (2005), and Kilbride (2007). Findings can be interpreted as representing the development of a CoP.

### Building a stroke team

A geographical SU was essential … a central hub for connecting people and facilitated networking opportunities. Having a base allowed people to form relationships (Focus group 1).

Staff named the unit “STEP” (Stroke Treatment for Every Person); this helped identify the stroke team. Staff deployed to STEP from different clinical areas and professional groups and their differing backgrounds and experiences meant that just being relocated together did not achieve team-working; as identified by early interprofessional disputes and territorial practices, particularly between nurses and physiotherapists. Attention was needed to build a team before people could work together collaboratively.

A number of ways were used. An operational infrastructure (Table I) provided a framework for ongoing activities. Interprofessional team projects presented the chance to get to know each other through actions other than direct patient care. Projects included creating a local stroke booklet, developing interprofessional patient records and organizing a Charity Ball. The weekly 1-hour STEP meetings, which were neither patient-focussed nor consultant-led, became a nucleus for teamwork. Meetings were voluntary and often had no pre-arranged agenda but were problem focussed. Initially difficult to persuade staff to take time away from patients, this became a protected slot when staff saw achievements from having time for service development.

#### Developing practice-based knowledge and skills in stroke

Transferred from neurology or elderly care settings, at the outset most staff had little stroke expertise. An interprofessional seminar series based on stroke guidelines was the starting point. Nurses experienced greatest difficulty taking time away from the ward to attend; a rota of therapists was drawn up to provide assistance on the unit to facilitate nursing attendance. This act in support of team education underpinned team development.

The education programme has been key, feels better because you know things. Knowledge and participation is the trick. (Nurse 8)

Additionally, a stroke coordinator was appointed and this experienced stroke nurse provided a role model for nurses and was a resource for the whole team.

As the SU became established, staff expressed positive comments about the unit as a learning environment; staff took advantage of everyday activities to enhance informal development of stroke knowledge and skills. With shared SU space and improved team relationships, learning from working alongside each other was a strong theme.

You may go and help a nurse reposition a patient … gives us the opportunity for both to ask and answer questions … (Therapist 14)

SU development processes thereby promoted experiential learning opportunities, facilitated personal growth of knowledge and skills and helped embed this way of working into everyday practice.

#### Recognizing and valuing the central role of the nurse in stroke

Nurses as the only professionals continually present on SUs are ideally positioned to act as a hub for team activity; this role can only be taken up with the prerequisite expertise to do so. SU nurses' previous non-specialist work with stroke patients meant initially many were unable to fulfil this role.

Before we used to do the caring, and we wouldn't make any plans or goals to achieve. We just nursed them to make them better. (Nurse 3)

Nurses described how focussing on stroke enabled them to develop domain-specific knowledge and expertise. With increased skills and knowledge in stroke care along with improving team-working, they developed from elderly care generalist to stroke specialist nurses, and began to claim a pivotal team role.

#### Establishing an organizational climate to support improvement

Clinical projects involving major service redesign require managerial assistance for success and sustainability; the appointment of a new senior manager and Therapy Head was invaluable for practitioners promoting the stroke agenda. The growing profile of the SU and staff within the hospital and externally supported stroke service improvement. Opportunities to promote the SU were actively sought by the STEP team; involvement in developing the SU and enthusiasm generated by being part of an improvement initiative helped drive plans into action.

Being asked to come up with ideas and then being allowed to run with them … It is so motivating (Therapist 13)

An interprofessional stroke committee was convened, combining senior management and SU clinicians. This became a medium to influence strategy and strategically linked the SU horizontally and vertically within the organization.

Taken altogether, these processes can be considered as representing development of a CoP.

### A Community of Practice

There are few documented examples of the development of healthcare CoPs, especially comprised of more than a single professional group. However, similarities between study findings and processes described in the literature provide an explanatory framework for this case study. The three elements of CoPs (Figure 1) are discussed in the light of the study findings.

### Domain

The domain focus was improving stroke care. The geographical nature of the SU was fundamental but proximity alone was not enough for practice development (Ferlie, 2006). Mutual engagement of staff was required, via participatory interaction. Establishing this domain defined the core purpose of staff actions, providing common ground for shared understanding as a basis for collaborative working (Wenger et al., 2002). This collective position and united vision helped overcome early difficulties as individuals learned to work together. It focussed formal learning and the evolving common body of knowledge. Bringing staff together in the domain was the starting point for the CoP.

The stroke domain provided a peg for raising the visibility of stroke within the Trust. As in this study, SUs are often based within elderly care; having designated space helped separate and promote the emerging specialist profile of stroke from the generalist elderly care setting. Stroke care as a specialism became recognized within the organization, this increased visibility was purposively strengthened by activities to raise the profile and value of stroke, including a formal celebrity unit opening (Kilbride et al., 2005; Kilbride, 2007). Wenger et al. (2002) suggested high level endorsement reinforces domain importance to managers, boosting their support.

Through the collective identity of the SU staff felt part of something meaningful, which contributed to development both of the CoP and individual professional identities. Definition of self and identity is negotiated through what we do; the stroke domain built a meaningful sense of shared identity that tied people beyond specific workplace exchanges (Wenger, 1998; Wenger et al., 2002).

### Community

A *community* is a group of people who care about a specific domain and through interaction in practice activity create the “social fabric of learning” (Wenger et al., 2002, p. 28). As the CoP developed, elements functioned synergistically, with centralization of the domain providing the base for developing the community. Individuals' joining as a community is both a form of action and an act of belonging, influencing not only what we do but how we interpret ourselves; knowing, belonging and doing are inseparable in a CoP (Wenger, 1998). Individual and group identities from being part of the SU contributed to a sense of belonging to a particular community (Wenger, 1998); staff felt part of something that mattered. Through regular interactions during patient interventions and goal planning, staff built relationships leading to a sense of community and development of social capital (Gersick et al., 2000; Whetten, 2001).

Social capital is the wealth or benefit that exists within a network of individuals (Lesser, 2000). Through making and keeping connections such as those described in our study findings, staff capitalized on shared endeavours. CoPs have been described as vehicles for generating social capital, by developing connections amongst practitioners and fostering relationships building mutual confidence and obligation (Lesser & Storck, 2001). Social capital is linked to behaviours like trust and respect. Weekly STEP meetings, for example, enabled members to show they could be trusted to act on agreed actions; staff working together in patient sessions learnt to consult each other and share knowledge. Relationships fostering interactions based on mutual trust and respect give people a sense of belonging and help bind members together within a social entity (Wenger et al., 2002). Development of professional relationships and social ties are important reasons why people stay within organizations (Cappelli, 2000); a key consideration for all institutions. Hence, creation of social capital during the development of this CoP may have been a seminal feature of this SU's success.

A key difference between a CoP and a conventional team lies in structure; CoPs are based on collegial relationships rather than hierarchical management structures featured in many conventional teams (Bate & Robert, 2002). The collegial nature of SU interprofessional relationships, including medical staff, was repeatedly illustrated. Practitioners are often poor at recognizing the management role in clinical change (Fitzgerald & Dopson, 2006); within CoPs it is seen as helping align clinical practice with political priorities (Wenger et al., 2002). Having managers in the stroke community strengthened links between organizational levels, extended the shared meaning of best stroke care in the Trust whilst reducing perceived distance between management and clinicians, it also flattened the local hierarchy (Coghlan & Brannick, 2005). Furthermore, the dual managerial and clinical responsibilities of the lead investigator provided opportunities to work horizontally and vertically in the Trust acting as a “boundary spanner”, a term applied to people with significant ties across boundaries and organizations (Rogers, 1995). Fitzgerald et al. (2002) describe this role as linking academic or expert worlds to the practitioner's, helping diffuse innovations and improve information flow.

Hence *domain* was intrinsic to *community* growth, and as care is largely socially mediated (Harrison, 2001), *community* was fundamental to *practice.*

### Practice

Practice is present in the community through mutual engagement of members in domain activity. Practice is described as the result of collective learning and reflective of social relations and shared community endeavour (Wenger, 1998). Practice activities presented ways to connect community members in pursuit of delivering best stroke care. For example, the team documentation was more than a record of patient information; reading other entries stimulated staff questions, thus creating learning opportunities. The weekly STEP meeting provided space for reflection on action, a key aspect of practice-based learning (Coghlan & Brannick, 2005). Space is important for staff to “internalise and shape processes of change” (Elsey & Lathlean, 2006, p. 171); failure to make time for regular meetings to encourage participation in structured decision-making can negatively impact on team-working and communication (Field & West, 1995).

The theme *Developing practice-based knowledge and skills in stroke* showed accumulation of stroke knowledge was instrumental in staff developing a sense of practice expertise. Practical knowledge involves what we know, what we do, and how we act (Gustavsson, 2004). This sense of *knowing* about practice can be developed and refined in CoPs, where shared understanding is core (Abrandt Dahlgren et al., 2004). Creating an identity through the stroke domain contributed to the growing sense of practice expertise in staff, especially for nurses. Until nurses had sufficient specialist skills and knowledge they were not able to play a full role in the community. This was significant; if one profession within a team lacks expertise, it can adversely affect their participation with other team members filling gaps (Anstey, 2003). As the nurses developed stroke expertise they took up a central community role strengthening their professional identity as specialist nurses. Thus learning had a transformational effect beyond improving the individual knowledge base: “learning transforms who we are and what we can do, it is an experience of identity… a process of becoming” (Wenger, 1998, p. 215).

Practice includes development and use of frameworks, tools, language and documents that help guide and build upon established knowledge (Lave & Wenger, 1991; Wenger et al., 2002). Operational infrastructure activities shown in Table I promoted a shared language, further strengthening the concept of learning as a social process. As practice is the enactment of stroke knowledge, the evidence base for stroke care at the Trust became rooted in practice through mutual engagement of community members in pursuit of the domain.

## DISCUSSION

National Stroke Guidelines recommends SU care (ISWP, 2008), but predominant focus on research outcomes has limited process-related knowledge. It has been suggested patients do better on SUs because staff work together to deliver good care (SUTC, 2007; Langhorne & Pollock, 2002); this is the first study to reveal practical processes required to develop such working relationships, in this case in the guise of a CoP. Whilst not a study aim, findings support the contention that CoPs are important in implementing evidence-based healthcare (Fitzgerald et al., 2006). That what was created was more than a team is indicated by a number of departures from “usual” team function, such as inclusion of a manager and collegial rather than hierarchical or functional relationships between members. Further, this CoP was fundamentally self-established, being composed of staff who originally worked in a dispersed service. Their own recognition of their *domain* and *practice* brought them together to form the basis of a *community.*

Findings are timely as the central role of CoPs has been identified in locally implementing evidence into practice (Barwick et al., 2009; Dopson & Fitzgerald, 2006). Seven UK evidence-based practice implementation studies found CoPs strongly influenced by professional affiliations, tended to be uniprofessional with marked group identities, and liaised poorly with neighbouring work groups; it appeared, “great effort is required to create a functioning multidisciplinary Community of Practice” (Dopson & Fitzgerald, 2006, p.10). By contrast, in this case study whilst interprofessional tensions were evident early, the shared purpose and focus of activities on improving stroke care seemed to lessen issues of professional jurisdiction shown to deter knowledge transfer between practitioner groups (Abbott, 1998). Interprofessional competition was decreased by focusing on patient care, with consequently increased give and take across professional boundaries (Egan & Jaye, 2009). This shift in focus was achieved during the “project” stages of this service development and for some time later, insofar as indicated by continued high performance in 2004, 2006 and 2008 NSSA rounds.

Research into healthcare CoPs is comparatively new and this study contributes the first empirical evidence of how this way of working with an interprofessional focus may be achieved. Findings support the recommendation that social perspectives of EBHC should be more widely recognized and utilized as it is through development of these social processes that global evidence is converted, accepted and used as local knowledge (Fitzgerald et al., 2006).

As with all research, this study has strengths and weaknesses. Representing processes developed in one time and place, readers must judge transferability of findings to other settings. The project focused on professional and organizational perspectives; stroke patients were involved but more user engagement would give greater confidence in the consumer-focus of the service. The Action Research approach enabled an in-depth view of how stroke care was implemented in a clinical setting, and the various methods allowed developments to be examined from multiple perspectives. The first author's “insider” role (Kilbride et al., 2005) provided access to information unlikely to have been so readily available to an external researcher. Whilst this arguably adds credibility to findings, the possibility of insider bias cannot be ignored. To this end, critical skills in self-awareness, sensitivity and reflexivity were important, along with supervision provided by other authors.

## CONCLUSION

This study set out to identify key factors which influenced outcomes; a number of elements acting synergistically were probably central. Some are well-known, such as the importance of top-down organizational engagement as well as bottom-up involvement. Aspects that were not anticipated include the need to pay attention to building and maintaining the team and its identity, valuing diversity and celebrating successes; importance of developing relationships, trust and social capital; the value of protected time for team members to reflect, plan and work together across professional boundaries, and making learning an everyday occurrence; the utility of vertical and horizontal organizational links. Benefit probably also accrued by fostering a research culture in clinical practice with academic collaboration to maximize practice-based learning.

Further research is needed to explore whether what was achieved can be replicated in different settings and with conditions other than stroke. However, findings clearly indicate that the creation of CoPs through the use of Action Research holds promise as a strategy to implement EBHC.
